# Hippocampal NLRP3 Inflammasome Drives Anxiety- and Depressive-Like Behaviors Following Drug-Induced Liver Injury

**DOI:** 10.7150/ijbs.128620

**Published:** 2026-09-03

**Authors:** Hao Zhang, Jia-Lu Zhuang, Rui Ma, Sheng Wang, Jing Huang, Hui-Xia Yang, Ming-Ming Hu, Xiao-Li Wei, Hai-Yuan Shen, Hui Fang, Wen-Ning Wu, Hua Wang

**Affiliations:** 1 Department of Oncology, the First Affiliated Hospital of Anhui Medical University, Hefei, 230022, PR China.; 2 Key Laboratory of Anti-inflammatory and Immune Medicine (Anhui Medical University), Ministry of Education, Hefei, 230032, PR China.; 3 Department of Pharmacology, School of Pharmacy, Anhui Medical University, Hefei, Anhui, 230032, PR China.; 4 Innovation and Entrepreneurship Laboratory for College Students, Anhui Medical University, Hefei, Anhui, 230032, PR China.; 5 Center for Scientific Research of Anhui Medical University, Anhui Medical University, Hefei, Anhui, 230032, PR China.

**Keywords:** drug-induced liver injury, anxiety- and depressive-like behaviors, inflammation, NLRP3 inflammasome

## Abstract

Drug-induced liver injury (DILI) is a considerable and potentially severe adverse reaction to medications. As drug abuse continues to rise, managing drug-induced liver toxicity has become a considerable challenge for clinicians. Depression is a chronic neurological disorder with a rising incidence globally in recent years, adding to the overall societal burden. Numerous clinical studies have suggested a link between DILI and mental illnesses, with inflammation potentially playing a mediating role. However, the exact mechanisms remain unclear. Here, we established a mouse model of DILI through acetaminophen (APAP) exposure and assessed the correlation between mental disorder and NLRP3 inflammasome. We found that DILI mice exhibited anxiety- and depressive-like behaviors, along with disrupted blood-brain barrier integrity and a significant increase in hippocampal NLRP3 inflammasome levels. *Nlrp3* knockout or hippocampal *Nlrp3* knockdown ameliorated DILI-induced anxiety- and depressive-like behaviors. Moreover, N-acetylcysteine, an agent against APAP-induced hepatotoxicity, inhibited NLRP3 inflammasome activation and alleviated anxiety- and depressive-like behaviors in DILI mice. These results indicate that the NLRP3 inflammasome might be a promising target for the treatment of patients with DILI who experience comorbid anxiety and depression.

## Introduction

Drug-induced liver injury (DILI) refers to a medical condition where unintended liver damage results from a drug or its metabolites during recreational or therapeutic use. As the primary organ responsible for drug metabolism, the liver is highly susceptible to injury caused by pharmacological agents. The pathogenesis of DILI is primarily driven by inflammation, which arises from biochemical and organelle stress, hepatocyte cell death, and the accumulation of drugs or their metabolites within the liver 1-3. Acetaminophen (APAP) is among the most widely used analgesic and antipyretic medications worldwide. However, excessive intake of APAP is a leading cause of DILI and may lead to acute liver failure 4-6. With the rising prevalence of drug abuse, DILI has become a major therapeutic challenge for clinicians globally, as it is linked to a range of systemic complications, including bile duct injury 7 and severe cutaneous adverse reactions such as skin rashes, facial edema, Stevens-Johnson syndrome, toxic epidermal necrolysis, and drug reaction with eosinophilia and systemic symptoms 8, 9.

In recent years, the concept of the liver-brain axis has gained attention, with growing evidence suggesting that liver diseases may be linked to cognitive deficits and mental disorders 10. Major depressive disorder (MDD), a common and recurrent disorder, is a leading global mental health challenge that affects approximately 4.4% of the population 11, 12. Patients with depression usually present with persistent low mood, poor appetite, and disrupted sleep, and in severe cases, they may engage in suicidal behavior. These symptoms not only seriously impact their quality of life but also place a substantial burden on society 13. Numerous clinical studies have demonstrated that patients with chronic liver diseases have a higher prevalence of depression than healthy individuals, with nearly one-third of patients with cirrhosis or hepatitis showing depressive symptoms 14, 15. Animal studies have further corroborated these findings, by reporting the development of depressive-like behaviors in mice with experimental chronic liver injury 16. Additionally, clinical studies have revealed that patients with DILI may experience anxiety, especially mild anxiety 17, and that depression is closely associated with APAP-induced liver injury 18. A similar phenomenon has been observed in animal experiments, where rats with DILI may exhibit anxiety-like behaviors accompanied by cognitive decline 19. Despite these observations, the mechanisms regulating the liver-brain axis and the association between DILI and anxiety- and depressive-like behaviors remain poorly understood. Therefore, a deeper understanding of the relationship between DILI and psychiatric symptoms, as well as their underlying mechanisms, is essential for developing novel therapeutic strategies.

Inflammasomes are multi-protein complexes consisting of an upstream sensor from the NOD-like receptor (NLR) family, the adapter protein ASC (apoptosis-associated speck-like protein containing a caspase-recruitment domain [CARD]), and the downstream effector cysteinyl aspartate specific proteinase-1 (caspase-1). Inflammasome activation has been implicated in cardiovascular and metabolic disorders, depression, Alzheimer's disease, and type 2 diabetes, among other diseases 20-22. Inflammasomes are activated by a variety of invasive pathogens and cellular stress signals, subsequently promoting the production and release of proinflammatory cytokines such as interleukin (IL)-1β and IL-18 23, 24. IL-1β, in particular, can trigger the proliferation of neuroinflammatory cells such as macrophages, microglia, and astrocytes, thereby promoting the progression of neuroinflammation 25. The NLR family, pyrin domain containing 3 (NLRP3) inflammasome, a widely studied member of the NLR family of proteins, plays a key role in various inflammatory responses. It also interacts with the nuclear factor-kappaB (NF-κB) signaling pathway, regulating IL-1β transcription and activity, which drives innate immunity and inflammation in the central nervous system (CNS) 26, 27. However, the role of the NLRP3 inflammasome in the interplay between DILI and anxiety- and depressive-like behaviors remains unclear.

In this study, we established a DILI model through APAP exposure and evaluated the interplay between DILI, anxiety- and depressive-like behaviors, and NLRP3 activation. Our findings demonstrate that the NLRP3 inflammasome plays a critical role in the development of anxiety- and depressive-like behaviors induced by DILI, underscoring its potential as a therapeutic target in clinical applications.

## Materials and Methods

### Animals

Male C57BL/6J mice (7-8 weeks of age, 22-25 g) were purchased from GemPharmatech Co., Ltd. *Nlrp3*^-/-^ mice were contributed by Jiang Wei's laboratory at the University of Science and Technology of China, and the generation and characterization of these mice have been previously described 28. All mice were housed under standard specific pathogen-free conditions with free access to water and food. The indoor temperature was controlled at 22 ± 2℃ and the relative humidity was 60%, and the light cycle was 12 h (8:00-20:00).

### Drug administration

Male C57BL/6J mice (7-8 weeks old, 22-25 g) were fasted overnight 29 and then intraperitoneally injected with 300 mg/kg APAP (S31044, Shanghai yuanye Bio-Technology Co., Ltd) dissolved in phosphate-buffered saline (PBS). Control mice were intraperitoneally injected with a similar volume of PBS 30. 300 mg/kg N-acetylcysteine (NAC) (S20137, Shanghai yuanye Bio-Technology Co., Ltd) was intraperitoneally injected 2 h before APAP injection 31.

### Sucrose preference test (SPT)

SPT was conducted in mice 24 h after APAP injection to assess the presence of a depressive-like state. Each mouse was housed in a single cage for 24 h. Two standard drinking bottles were used, one with pure water and the other with an equal volume of 1% sucrose. The position of the water jugs was changed every 12 h to avoid side preferences. Sucrose and water intake were measured at the end of the SPT, and the sucrose preference rate was calculated as sucrose intake / (sucrose intake + water intake) × 100%. A reduced sucrose preference indicates a diminished sense of pleasure and a potential depressive-like behavior.

### Elevated plus maze (EPM)

The EPM was conducted to assess anxiety-related behavior in mice. Before the experiment, the mice were placed in the EPM environment, allowing them to adapt to it for 2 h. After cleaning the maze, the mice were placed in its central area, and the distance traveled as well as the time spent in the open arms over the next 5 min were recorded. Between assessments, the maze was cleaned with 75% ethanol to eliminate any potential olfactory cues. Shorter travel distances and longer latencies to enter the open arms in the EPM suggest anxiety-like behavior.

### Open-field test (OFT)

The OFT was performed to evaluate autonomous locomotor behavior. Before the experiment, the mice were placed in the open-field apparatus which consisted of nine equal squares and allowed to adapt for 2 h. After cleaning the device, the mice were placed in the central area for 2 min, and then the movement distance and the time spent in the center area were recorded over the next 3 min. After the experiment, the OFT apparatus was cleaned with 75% ethanol to avoid the interference of odor signals. Reduced movement distance and decreased time spent in the center area indicate anxiety- and depressive-like behavior.

### Forced swimming test (FST)

The FST was further conducted to evaluate the presence of a depressive-like state. The experimental device was composed of a transparent bucket filled with water at a temperature of 23 ± 0.3℃. The water depth was adjusted to prevent the mice from using their legs for support or touching the bottom of the apparatus. At the beginning of the experiment, the mice were placed in the bucket, allowing them to move freely for 2 min, and then their immobility time was recorded over the following 4 min. For subsequent testing, the water was replaced and adjusted again to 23 ± 0.3℃. An increased time of immobility is indicative of a depressive-like behavior.

### Biochemical assays

Mouse peripheral blood was collected and centrifuged to obtain serum for analysis. We used a Mindray automated biochemical analyzer (BS-350E) to test serum alanine aminotransferase (ALT) and aspartate aminotransferase (AST) levels.

### Hematoxylin and Eosin (H&E) staining

Liver tissue was fixed with 4% paraformaldehyde, embedded in paraffin, and cut into 4-μm sections. After xylene dewaxing and rehydration in graded ethanol, routine staining with hematoxylin and eosin was performed. Images of liver necrosis were taken under light microscopy.

### Blood-brain barrier (BBB) permeability assay

Mice injected with PBS or APAP were intravenously injected with 1 mL/kg of 2% Evans blue (E2129, Sigma) dissolved in saline 32. The mice were then anesthetized and perfused with 0.9% saline, and 100 mg of brain homogenate was incubated with 1 mL formamide at 55℃ for 24 h to extract the Evans blue dye. The formamide/Evans blue mixture was centrifuged and the absorbance was measured at 610 nm in a microplate reader 33. A separate set of mice was perfused with 4% paraformaldehyde after flushing with 0.9% saline. Mouse brains were then quickly removed, post-fixed in 4% paraformaldehyde for 4 h, and placed in 30% sucrose solution at 4℃ for cryoprotection. Sections (14 μm thick) were obtained via cryosectioning and mounted on gelatin-coated glass slides. After three 5 min washes in PBS, a Tissue Gnostics Tissue FAXS Plus ST system equipped with a 620 nm laser was used to examine the brain sections 32.

### Western blotting

After concluding the behavioral tests, the mice were sacrificed under anesthesia and brain tissue was collected. Hippocampal and cortical tissues were treated with lysis buffer containing a protease and phosphatase inhibitor mixture (P1050, Beyotime) for 30 min on ice and homogenized, and the supernatant was separated by centrifugation at 12,000 rpm for 15 min at 4℃. Protein concentration was determined using a BCA protein assay kit (P0010S, Beyotime). Equal amounts of protein were separated by electrophoresis, transferred to PVDF membranes, and blocked for 2 h to inhibit non-specific antibody binding. After rinsing at room temperature, the PVDF membranes were incubated overnight at 4℃ with primary antibodies (anti-ZO-1, 1:1,000, AF5145, Affinity; anti-Occludin, 1:1,000, DF7504, Affinity; anti-NLRP3, 1:1,000, 15101S, Cell Signaling Technology; anti-cleaved-caspase-1, 1:500, AF4005, Affinity; anti-ASC, 1:1,000, 67824S, Cell Signaling Technology; anti-NF-κB, 1:1,000, 8242S, Cell Signaling Technology; anti-p-NF-κB, 1:1,000, 3033S, Cell Signaling Technology; anti-CYP2E1, 1:1,000, Ab28146, Abcam). After rinsing at room temperature, horseradish peroxidase (HRP)-coupled secondary antibodies were added for 2 h at room temperature. Finally, the membranes were rinsed and visualized in a gel imager after reacting with chemiluminescent substrate (D046, Bridge Bio) for 5 min.

### Quantitative real-time PCR analysis

After concluding the behavioral tests, the mice were sacrificed under anesthesia and their brains removed. Total RNA was extracted from each sample using an RNAeasy™ Animal RNA Isolation Kit (R0027, Beyotime) as per the kit's instructions. cDNA synthesis was performed using a PrimeScript First Strand cDNA Synthesis Kit (RR036A, Takara) applying standard cDNA amplification methods, with GAPDH as internal control. The following specific primers were used: NLRP3 (forward: 5-GCTGCGATCAACAGGCGAGAC-3, reverse: 5-CCATCCACTCTTCTTCAAGGCTGTC-3); Caspase-1 (forward: 5-ATACAACCACTCGTACACGTCTTGC-3, reverse: 5-TCCTCCAGCAGCAACTTCATTTCTC-3); ASC (forward: 5-GGACGGAGTGCTGGATGCTTTG-3, reverse: 5-CATCTTGTCTTGGCTGGTGGTCTC-3); GAPDH (forward: 5-AGGTCGGTGTGAACGGATTTG-3, reverse: 5-TGTAGACCATGTAGTTGAGGTCA-3).

### Enzyme-linked immunosorbent assay (ELISA)

After concluding the behavioral tests, the mice were sacrificed under anesthesia and brain and serum samples were collected. Using ELISA kits from ELK Biotechnology, the levels of IL-1β (ELK1271), IL-18 (ELK2269), IL-6 (ELK1157), and tumor necrosis factor-α (TNF-α) (ELK1387) in brain homogenates and cortisol (ELK0599) in serum were measured according to the manufacturer's protocols.

### Immunofluorescence

After completing the behavioral tests, the mice were anesthetized and transcardially perfused with 0.9% saline. The brains were removed, fixed in 4% paraformaldehyde for 4 h, and transferred to 30% sucrose in PBS overnight. Brain tissues were cut into 14-μm sections in a cryostat, washed three times in PBS, blocked at room temperature for 2 h, and incubated with anti-Iba-1 (1:100, Ab283319, Abcam), anti-CD68 (1:50, Ab303565, Abcam) or anti-NLRP3 (1:250, GB114320, Servicebio) primary antibodies at 4℃ overnight. The slices were then washed with PBS, incubated with suitable fluorescently labeled secondary antibodies at room temperature for 1 h, and counterstained with DAPI. After mounting, images were collected under epifluorescence microscopy (Leica, Germany) and analyzed using ImageJ software.

### Hippocampus-specific knockdown of *Nlrp3* and lateral ventricle-specific overexpression of* Zo-1*

Adeno-associated virus (AAV) vectors were used to knock down NLRP3 (*Nlrp3*-shRNA) in the hippocampus and overexpress ZO-1 (AAV-*Zo-1*) in the lateral ventricle of mice. The *Nlrp3*-shRNA and Control-shRNA constructs were cloned into pHBAAV-U6-MCS-CMV-EGFP and confirmed by sequencing (Hanbio Tech, Shanghai, China). The AAV-*Zo-1* (CRISPR activation) and AAV-eGFP constructs were cloned into PFD-rAAV-U6-sgRNA-mini-EF1a-SV40 NLS-dCasMINI-V4-2XSV40 NLS-NFZ-P2A-EGFP-WPREs and confirmed by sequencing (BrainVTA, China). Four-week-old C57BL/6J male mice were anesthetized, and the scalp was clipped to expose the skull. For hippocampal knockdown, bilateral microinjections were conducted to target the hippocampus (-1.6 mm anteroposterior (AP), ±1.8 mm mediolateral (ML), -1.6 mm dorsoventral (DV)) with either Control-shRNA (AAV2/9, 1.4 × 10^12^ vg/mL, Interference sequence: 5'-TTCTCCGAACGTGTCACGTAA-3') or *Nlrp3*-shRNA (AAV2/9, 1.2 × 10^12^ vg/mL, Interference sequence: 5'-CCGGCCTTACTTCAATCTGTT-3'). For each injection, 1.5 μL of the vector was infused at a rate of 0.25 μL/min 34. For lateral ventricle overexpression, mice received bilateral intraventricular (-0.2 mm AP, ±1.0 mm ML, -2.0 mm DV) injections of AAV-eGFP (AAV2/9, 5.95 × 10^12^ vg/mL, sgRNA sequence: 5'-GTGTAGTTCGACCATTCGTG-3') or AAV-*Zo-1* (AAV2/9, 5.71 × 10^12^ vg/mL, sgRNA sequence: 5'-CGGTCATAATCGACTGCAAATAG-3'). For each injection, 2.5 μL of the vector was infused at a rate of 0.25 μL/min. After the procedure, the mice were placed on a heating pad until they recovered. After 4 weeks, the efficacy of transduction was assessed by immunofluorescence and western blotting.

### Liquid chromatography-tandem mass spectrometry (LC-MS/MS)

Brain tissue was homogenized with three-fold volumes of 70% methanol-water solution (v/v). An aliquot of 500 µL of the homogenate was mixed with 50 µL of internal standard (APAP-d_3_, 1 µg/mL in 70% methanol-water solution) and 1 mL of acetonitrile was added to precipitate proteins. After centrifugation, the supernatant was evaporated to near dryness under a stream of nitrogen. The residue was reconstituted in 150 µL of 70% methanol-water solution, centrifuged, and the supernatant was transferred into a brown vial with a lined insert for LC-MS/MS analysis. Chromatographic separation was performed on a Waters HSS T3 column (100 mm × 2.1 mm, 1.8 µm) by using mobile phase A (acetonitrile) and mobile phase B (water containing 0.1% formic acid). Linear gradient elution was applied as follows: 0-1 min, 90% B; 2 min, 10% B; 3.5 min, 10% B; 3.6 min, 90% B; and 5.0 min, 90% B. The flow rate was 0.20 mL/min, the column temperature was maintained at 40 °C, and the injection volume was 5 µL. Orbitrap ultra-high resolution mass spectrometry was conducted by using a heated electrospray ionization (HESI) source in positive ion mode. The spray voltage was set to +4.50 kV, capillary temperature to 320 °C, auxiliary gas heater temperature to 200 °C, sheath gas (nitrogen) pressure to 350 kPa, auxiliary gas pressure to 70 kPa, and sweep gas pressure to 3.5 kPa. Data acquisition was performed in Full MS/dd-MS2 mode, with a full MS scan range of m/z 75-1000, resolution of 70,000 for MS1 and 17,500 for MS2, and normalized collision energies (NCE) of 20%, 30%, and 50%.

### Statistical analysis

Prism 8.0 for Windows (GraphPad software, USA) was used for statistical analysis. Student's *t*-test was used to compare differences between two groups, and one-way analysis of variance (ANOVA) was used to compare differences between multiple groups. All data were expressed as mean ± SEM, and P < 0.05 was considered significant.

## Results

### DILI induces anxiety- and depressive-like behaviors in mice

To investigate the effects of DILI on anxiety- and depressive-like behaviors, we first conducted intraperitoneal injection of 300 mg/kg of APAP or PBS in mice (Figure 1A). Compared with the control group, APAP exposure resulted in a significant increase in serum ALT and AST levels (Figure 1B). Pathological analysis of liver tissues revealed severe damage to hepatocyte structure in APAP-treated mice. Additionally, hepatocytes surrounding the hepatic sinusoids exhibited varying degrees of necrosis and degeneration, accompanied by extensive infiltration of inflammatory cells (Figure 1C). These findings suggested that APAP exposure successfully induced DILI.

Then, DILI-induced anxiety- and depressive-like behaviors were assessed using the EPM, OFT, SPT, and FST. In the EPM, the distance traveled and the time spent in the open arms of the maze were significantly reduced in DILI model mice compared to the control group (Figure 1D-E). In the OFT, the time spent in the center area and the distance traveled in the open field were also noticeably decreased in DILI mice (Figure 1F-G). Additionally, a remarkable decrease in sucrose preference in the SPT and a noticeable increase in immobility time in the FST were observed in the DILI model group compared with the control group (Figure 1H-I). These results suggested that DILI elicited anxiety- and depressive-like behaviors in mice. Previous studies have suggested that serum cortisol levels may be associated with the development of anxiety and depressive disorders 35. We also found that serum cortisol levels were markedly elevated after APAP exposure as compared to control mice (Figure 1J). These results highlighted a potential association between DILI and neurobehavioral alterations.

### DILI disrupts the BBB and activates microglia

To further determine the mechanism by which DILI induces anxiety- and depressive-like behaviors in mice, we first evaluated the integrity of the BBB, a critical structure that maintains brain homeostasis by regulating the exchange of solutes between the CNS and the systemic circulation, and preventing the entry of harmful substances into the brain. Evans blue, a dye with high affinity for albumin, was utilized to assess BBB integrity. Normally, plasma albumin cannot pass through the BBB. However, when the BBB is compromised, albumin-bound Evans blue can penetrate into the brain 33, 36. Following intravenous tail vein injection, Evans blue markedly accumulated in the brains of DILI but not control mice (Figure 2A-B). Tight junctions (TJs) between endothelial cells in the CNS are critical to maintaining the integrity of the BBB 37. Therefore, we measured the expression of TJ-related proteins such as ZO-1 and Occludin in the cortices of DILI and control mice using western blotting. The results showed that APAP exposure significantly reduced TJ protein expression in the brain (Figure 2C), indicating that the BBB is disrupted following DILI.

Microglia serve as the primary CNS-resident immune cells. They act as the first line of defense against pathogens and injury, thus playing a crucial role in maintaining immune homeostasis in the CNS 38. To assess microglial activation, we detected the expression of Iba-1 and CD68 by immunofluorescence in the hippocampus of mice 39. The results showed that the expression of Iba-1 and CD68 was increased in DILI mice compared to control mice. Accordingly, in the control group microglial cells were branched and exhibited a small soma area, indicative of a resting state. In contrast, in DILI mice, the microglia displayed an amoeboid morphology, swollen cell bodies, shortened protrusions, and a notable increase in cell numbers, suggestive of an activated state (Figure 2D, Figure S1A). These results suggested that DILI disrupted the BBB and activated microglia in the brain.

### DILI activates hippocampal NF-κB/NLRP3 inflammasome signaling

In recent years, the role of NLRP3 inflammasome in the brain's innate immune response has received increasing attention due to its relatively high activity in microglia. Notably, microglia have been identified as the primary cells expressing NLRP3 in clinical models of depression 40. To investigate the role of NLRP3 inflammasome signaling in DILI-induced anxiety- and depressive-like behaviors, we first assessed the mRNA and protein levels of NLRP3 inflammasome components in the hippocampus. A remarkable upregulation of NLRP3, ASC and caspase-1 was observed in the hippocampus of DILI mice compared to control animals (Figure 3A-C). Concurrently, immunofluorescence staining results for Iba-1 and NLRP3 revealed that microglia are the primary cellular source of NLRP3 in the hippocampus (Figure S1B). Furthermore, DILI caused a significant increase in p-NF-κB/NF-κB ratio and in the levels of proinflammatory cytokines such as IL-1β, IL-18, TNF-α, and IL-6 (Figure 3D-F). To delineate the liver-brain inflammatory axis in DILI, we first employed LC-MS/MS to assess whether APAP could enter brain tissue. N-Acetyl-4-benzoquinone imine (NAPQI) is a highly reactive metabolite of APAP generated predominantly through cytochrome P450 2E1 (CYP2E1)-mediated oxidation 41. Neither APAP nor NAPQI was detectable in brain tissue, and hippocampal CYP2E1 expression was not significantly altered (Figure S2). Together, these findings indicated that the behavioral changes associated with DILI were unlikely to result from direct central exposure to APAP or its reactive metabolite. With this confounder excluded, we turned to the inflammatory response. Previous studies have established that the NF-κB signaling pathway is activated by microbial components or endogenous cytokines 42, and that APAP-induced liver injury correlates strongly with increased inflammation 43. We therefore examined cytokine changes in liver tissue and systemic circulation. Results revealed that hepatic and systemic proinflammatory factors were significantly upregulated by DILI (Figure S3). These findings suggested that DILI activated NLRP3 inflammasome signaling in the brain via liver-mediated cytokines, highlighting a potential mechanistic link between liver injury and anxiety- and depressive-like behaviors.

### *Nlrp3* knockout relieves DILI-induced anxiety- and depressive-like behaviors

To further assess the potential role of *Nlrp3* in DILI-induced anxiety- and depressive-like behaviors, we intraperitoneally injected 300 mg/kg APAP or PBS into both *Nlrp3*^-/-^ and wild-type mice fasted for 16 h, and performed behavioral tests 24 h post-injection (Figure 4A). *Nlrp3* knockout had no effect on sucrose preference in the SPT or immobility time in the FST, but ameliorated DILI-induced anhedonia (reduced sucrose preference) and behavioral despair (increased immobility time) in mice (Figure 4B-C). Similarly, *Nlrp3* knockout reversed DILI-induced reductions in both locomotion distance and the time spent in the open arms of the EPM and the central area in the OFT, while *Nlrp3* knockout had no effect on these parameters in PBS-injected mice (Figure 4D-G). These findings indicated that *Nlrp3* knockout markedly relieved anxiety- and depressive-like behaviors in DILI mice.

### Hippocampal *Nlrp3* knockdown ameliorates DILI-induced anxiety- and depressive-like behaviors, with BBB disruption as an upstream trigger

To further elucidate the role of *Nlrp3* in anxiety- and depressive-like behaviors caused by DILI, we successfully knocked down hippocampal *Nlrp3* expression by AAV-*Nlrp3*-shRNA microinjection. Four weeks post-injection, the mice were intraperitoneally injected with 300 mg/kg APAP, and behavioral tests were conducted 24 h later (Figure 5A-C). Compared with the control group, DILI significantly induced a decrease in sucrose preference and prolonged immobility time in the FST, while hippocampal *Nlrp3* knockdown markedly inhibited these effects (Figure 5D-E). Moreover, hippocampal *Nlrp3* knockdown reversed the reductions in distance and time spent in the open arms or central area in the EPM and OFT, while control shRNA infusion had no influence on them (Figure 5F-I). In addition, *Nlrp3* knockdown reduced circulating cortisol levels in DILI mice, and suppressed the upregulation of hippocampal ASC and caspase-1 protein levels induced by DILI (Figure 5J-K). These results highlighted the pivotal role of hippocampal NLRP3 in mediating anxiety- and depressive-like behaviors associated with DILI.

### Targeted BBB protection alleviates DILI-induced anxiety- and depressive-like behaviors

To verify the causal relationship between BBB disruption and NLRP3 activation, we first conducted time-course analyses, which revealed that BBB disruption preceded NLRP3 upregulation (Figure S4). To confirm this temporal precedence, we achieved specific BBB protection through lateral ventricular injection of AAV-*Zo-1*. Four weeks after viral injection, the DILI model was induced, followed by behavioral tests (Figure 6A-C). Results demonstrated that AAV-*Zo-1* significantly alleviated DILI-induced anxiety- and depressive-like behaviors and suppressed the associated NLRP3 upregulation (Figure 6D-J). Collectively, these results provided strong evidence that BBB disruption was an upstream event leading to NLRP3 activation and neurobehavioral changes in the course of DILI.

### NAC relieves DILI-induced anxiety- and depressive-like behaviors and inhibits NLRP3 inflammasome activation in mice

To further confirm the role of NLRP3 inflammasome in DILI-induced anxiety- and depressive-like behaviors, we intraperitoneally administered NAC, the only clinically approved antidote for APAP-induced hepatotoxicity, at a dose of 300 mg/kg to mice 2 h prior to APAP injection, and behavioral tests were conducted 24 h post-APAP injection (Figure 7A) 31. We found that NAC pretreatment significantly attenuated the increase in serum ALT and AST levels caused by DILI, and DILI-related hepatocyte necrosis and degeneration were also reduced following NAC administration (Figure 7B-C), indicating that NAC effectively mitigated APAP-induced liver injury.

Meanwhile, NAC pretreatment significantly increased the locomotion distance and time spent in the open arms and center area in the EPM and OFT (Figure 7D-G), restored sucrose preference (Figure 7H) and reduced the immobility time in the FST (Figure 7I). In addition, NAC pretreatment markedly downregulated the levels of cortisol in DILI mice (Figure 7J). These findings confirmed that NAC mitigated DILI and also counteracted the anxiety- and depressive-like behaviors caused by DILI.

Finally, we found that NAC pretreatment markedly reduced the circulating levels of IL-1β, IL-6 and TNF-α and inhibited the upregulation of hippocampal NLRP3, ASC and caspase-1 in DILI mice (Figure 7K, Figure S5). Taken together, these results underscored the critical role of liver-brain crosstalk in shaping anxiety- and depressive-like behaviors following DILI and highlighted the significance of hippocampal NLRP3 activity in mediating these effects.

## Discussion

This study demonstrated that DILI induces anxiety- and depressive-like behaviors in mice by impairing BBB function and activating central NF-κB/NLRP3 inflammasome signaling, as global *Nlrp3* knockout, hippocampal *Nlrp3* knockdown, and BBB protection all mitigated these behavioral changes. Moreover, NAC, a pharmacological intervention to counteract DILI, effectively reduced anxiety- and depressive-like behaviors and suppressed NLRP3 inflammasome activation. These findings suggest that the NLRP3 inflammasome plays a critical role in DILI-induced anxiety- and depressive-like behaviors.

In recent years, interorgan and tissue crosstalk has gained recognition as a crucial mechanism for maintaining homeostasis and facilitating adaptation to disease 44. As the largest internal organ, the liver performs many essential functions, including immune defense, detoxification, and nutrient metabolism. Its precise coordination with other organs is crucial for systemic homeostasis. A large number of clinical studies have highlighted the extrahepatic complications of acute and chronic liver diseases, as well as their associations with other systemic conditions 45.

DILI remains a leading cause of acute liver failure in western countries 46. APAP, a widely used analgesic and antipyretic, is a common cause of DILI due to its hepatotoxicity at high doses, often leading to severe outcomes such as liver transplantation or death 47, 48. Depression, a widespread mental illness characterized by a depressed mood, loss of interest, impaired cognitive function, and sleep and appetite disturbances, is a major contributor to global disability and disease burden 49, 50. However, in recent years, research on the relationship between DILI and anxiety- and depressive-like behaviors has largely remained at the clinical level 17, 18, 51, hence, the underlying mechanisms remain poorly understood. To address this gap, we established a mouse model of DILI through APAP exposure and characterized through behavioral tests the development of anxiety- and depressive-like behaviors in these mice. Our findings provide experimental evidence supporting the hypothesis that DILI can trigger anxiety- and depressive-like behaviors.

The BBB is a dynamic interface composed of brain microvascular endothelial cells, serving as both a mechanical and functional barrier that regulates the transport of substances between the blood and the brain. As an essential protective structure, its dysfunction has been implicated in various CNS disorders, including Alzheimer's disease and epilepsy 52. Emerging evidence suggests that liver injury can disrupt the BBB, contributing to cognitive and neuropsychiatric impairments. For instance, elevated levels of serum arginase in both liver injury patients and mice subjected to hepatic ischemia-reperfusion correlate with cognitive decline 53, 54. Arginase is highly expressed in hepatocytes. Following liver damage, excessive arginase release compromises BBB integrity by depleting arginine, a key factor in endothelial cell health, through its conversion into urea and ornithine 55. Based on these findings, we hypothesized that anxiety- and depressive-like behaviors caused by DILI are linked to BBB disruption resulting from liver damage. This hypothesis was supported by empirical data, which revealed a strong association between DILI-induced anxiety- and depressive-like behaviors and BBB dysfunction.

Growing evidence shows that microglia, the CNS-resident immune cells, are not only essential for maintaining immune homeostasis and regulating neuronal activity under physiological conditions, but also play a crucial role in the pathophysiology of depression 56, 57. Microglia are self-renewing myeloid cells derived from the yolk sac, accounting for about 10% of brain cells. They are considered CNS-specific immune cells, influencing CNS development, injury response, and repair 58. In response to CNS damage or infection, microglia undergo extensive morphological and functional changes upon activation of pattern recognition receptors (PRRs) on their surface, which recognize damage-associated molecular patterns (DAMPs) and pathogen-associated molecular patterns (PAMPs). This activation upregulates the expression of neuroinflammatory signaling genes and their receptors, thereby neutralizing the ongoing inflammatory process 59-61. In the present study, DILI induces extensive microglial activation across the hippocampal CA1, DG and CA3 subregions. Activated microglia may contribute to neuronal dysfunction not only through the direct release of inflammatory mediators but also through interactions with other glial cells. In support of this possibility, Hu et al. demonstrated that microglial NLRP3 inflammasome activation induces neurotoxic reactive astrocytes via a caspase-1-dependent neuroinflammatory pathway, thereby promoting neuronal dysfunction and depression-like behaviors 62. Although microglial activation was observed throughout multiple hippocampal subregions, we chose to focus our investigation on the CA3 subregion. As a critical hub in hippocampal information processing, the CA3 area possesses an extensive recurrent collateral network that underpins essential functions such as pattern completion 63. Notably, alterations in CA3 neuronal activity have been directly linked to depression-like phenotypes 64. Thus, by concentrating on CA3, we aimed to elucidate the critical role of the liver-brain axis and microglial activation in mediating anxiety- and depressive-like behaviors in the setting of liver injury.

PRRs play a pivotal role in initiating inflammatory signaling cascades within microglia. These receptors encompass several families, including Toll-like receptors, C-type lectin-like receptors, nucleotide-binding domain leucine-rich repeat containing receptors, retinoic acid-inducible gene (RIG)-I-like receptors, absent in melanoma 2 protein (AIM2)-like receptors, and cytosolic DNA sensors 65. Different types of closely related DAMPs can activate these PRRs to induce microglia to release proinflammatory cytokines. This process involves multiple signaling pathways, including key inflammatory cascades involving glycogen synthase kinase-3, NF-κB, and NLRP3 66, 67. Upon activation, the NLRP3 inflammasome facilitates the cleavage of pro-caspase-1 into its active form, which subsequently mediates the maturation and release of proinflammatory cytokines such as IL-1β and IL-18, thereby driving neuroinflammatory processes 62. Dysregulated NLRP3 inflammasome activation has been implicated in various pathological conditions, including chronic inflammatory disorders, metabolic syndromes, cardiovascular diseases, and neurodegenerative disorders 20, 21, 68. However, the role of the NLRP3 inflammasome in anxiety- and depressive-like behaviors caused by DILI remains poorly understood. Emerging evidence suggests a positive correlation between NLRP3 inflammasome activation and depression-like behavior in stress-induced depression models, highlighting the potential role of NLRP3 in the pathogenesis of depression 69-71. This evidence supports our hypothesis that the NLRP3 inflammasome may be involved in the development of anxiety- and depressive-like behaviors caused by DILI.

The hippocampus, a key component of the limbic system, has been extensively studied in depression research due to its structural and functional characteristics. Anatomically, it maintains extensive connections with emotion-related brain regions. Functionally, it contains a high density of glucocorticoid receptors, which allow the hippocampus to detect and respond to stress hormones like cortisol, as well as glutamatergic synapses that contribute to stress-induced neuroplasticity and influence the regulation of the hypothalamic-pituitary-adrenal axis. These features render the hippocampus particularly vulnerable to stress-induced alterations, suggesting that inflammatory processes may manifest in this region prior to other brain areas 72-74. To evaluate this hypothesis, we detected the expression of NLRP3 inflammasome-related genes in the hippocampus of mice with APAP-induced DILI. A significant upregulation of NLRP3, ASC and caspase-1 mRNA and protein levels, along with a marked elevation in proinflammatory cytokines, including IL-1β, IL-18, IL-6, and TNF-α, indicated that DILI triggers NLRP3 inflammasome assembly in the hippocampus, thus driving neuroinflammatory responses and promoting the development of anxiety- and depressive-like behaviors. Further substantiating our hypothesis, genetic ablation of NLRP3 significantly attenuated the manifestation of anxiety and depressive-like phenotypes in DILI mice. Subsequently, we performed stereotactic AAV-*Nlrp3*-shRNA injection to specifically knock down NLRP3 expression in the hippocampus. Notably, this targeted NLRP3 suppression substantially ameliorated anxiety- and depressive-like behaviors caused by DILI. These findings strongly suggest that hippocampal inflammatory responses mediated by the NLRP3 inflammasome play a pivotal role in the pathogenesis of anxiety- and depressive-like behaviors associated with DILI.

NAC is an acetyl derivative of the amino acid cysteine that has been used as an antidote to APAP poisoning since 1970 due to its potent antioxidant and anti-inflammatory properties 75, 76. Excessive APAP is metabolized by cytochrome P450 isoenzymes into the highly reactive intermediate metabolite NAPQI, which covalently binds to hepatocyte proteins to disrupt mitochondrial function and cause cell damage 77. As a precursor to glutathione (GSH), primarily synthesized in the liver, NAC mitigates this damage by indirectly aiding in the neutralization of reactive oxygen species and peroxynitrite, while also enhancing and restoring mitochondrial GSH levels 78. To further elucidate the crosstalk between liver and brain, we administered NAC to alleviate DILI. Besides mitigating liver injury, NAC supplementation reduced anxiety- and depressive-like behaviors and downregulated hippocampal NLRP3 inflammasome levels. These findings suggest the existence of a liver-brain axis linking liver injury to anxiety- and depressive-like behaviors. Our findings suggest that major proinflammatory factors released from the damaged liver enter the CNS via the circulation, leading to neuroinflammation. However, the exact mechanisms through which the liver-brain axis regulates DILI-related anxiety- and depressive-like behaviors remain unclear, warranting further investigation.

In summary, our study suggests the presence of a liver-brain axis, demonstrating that DILI can disrupt the BBB and induce the emergence of anxiety- and depressive-like behaviors. The NLRP3 inflammasome may be critically involved in the pathogenesis of these conditions, potentially serving as a novel therapeutic target for DILI patients who experience comorbid anxiety and depression.

## Supplementary Material

Supplementary figures.

## Figures and Tables

**Scheme 1:**
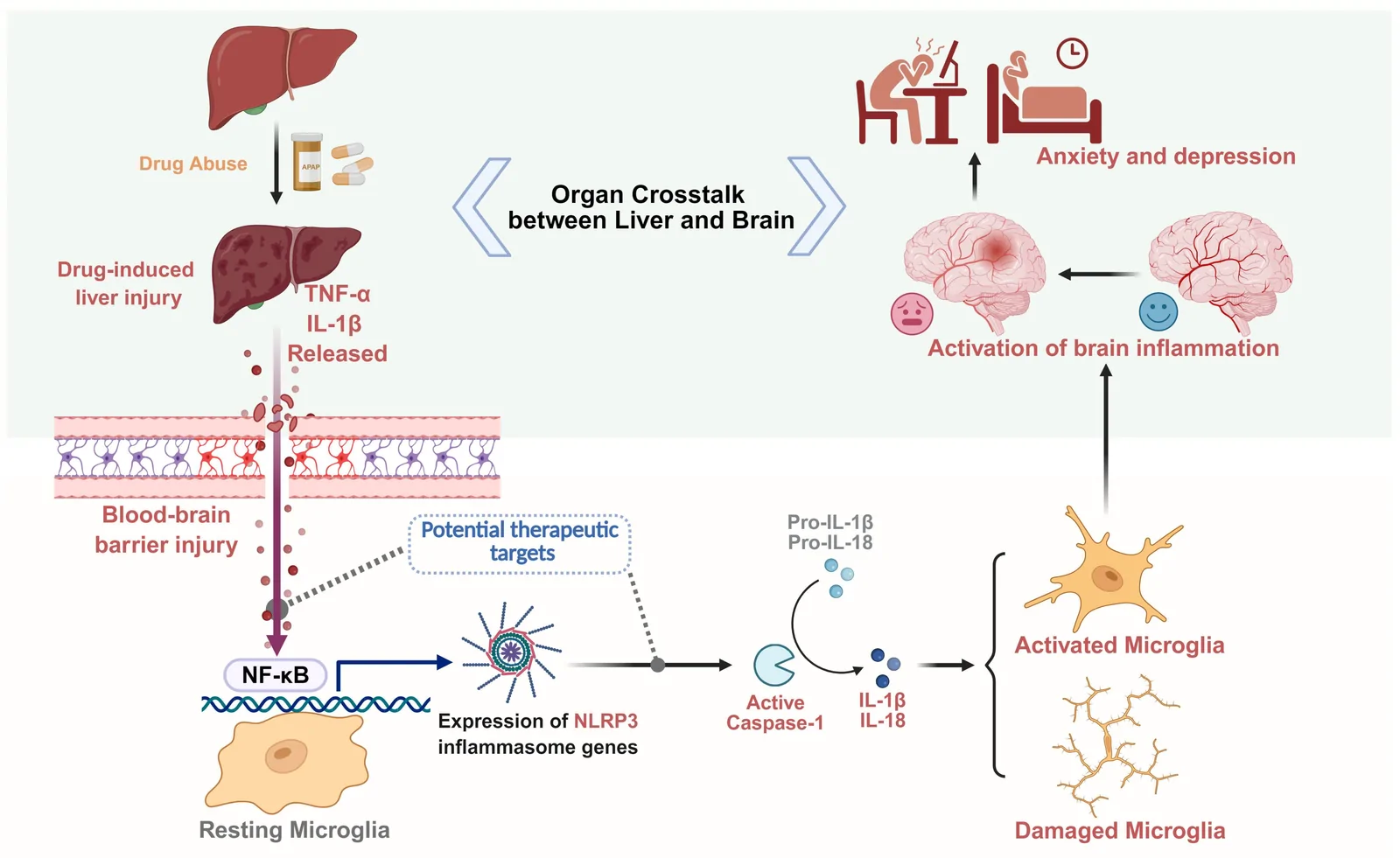
DILI induces anxiety- and depressive-like behaviors through the NLRP3 inflammasome.

**Figure 1:**
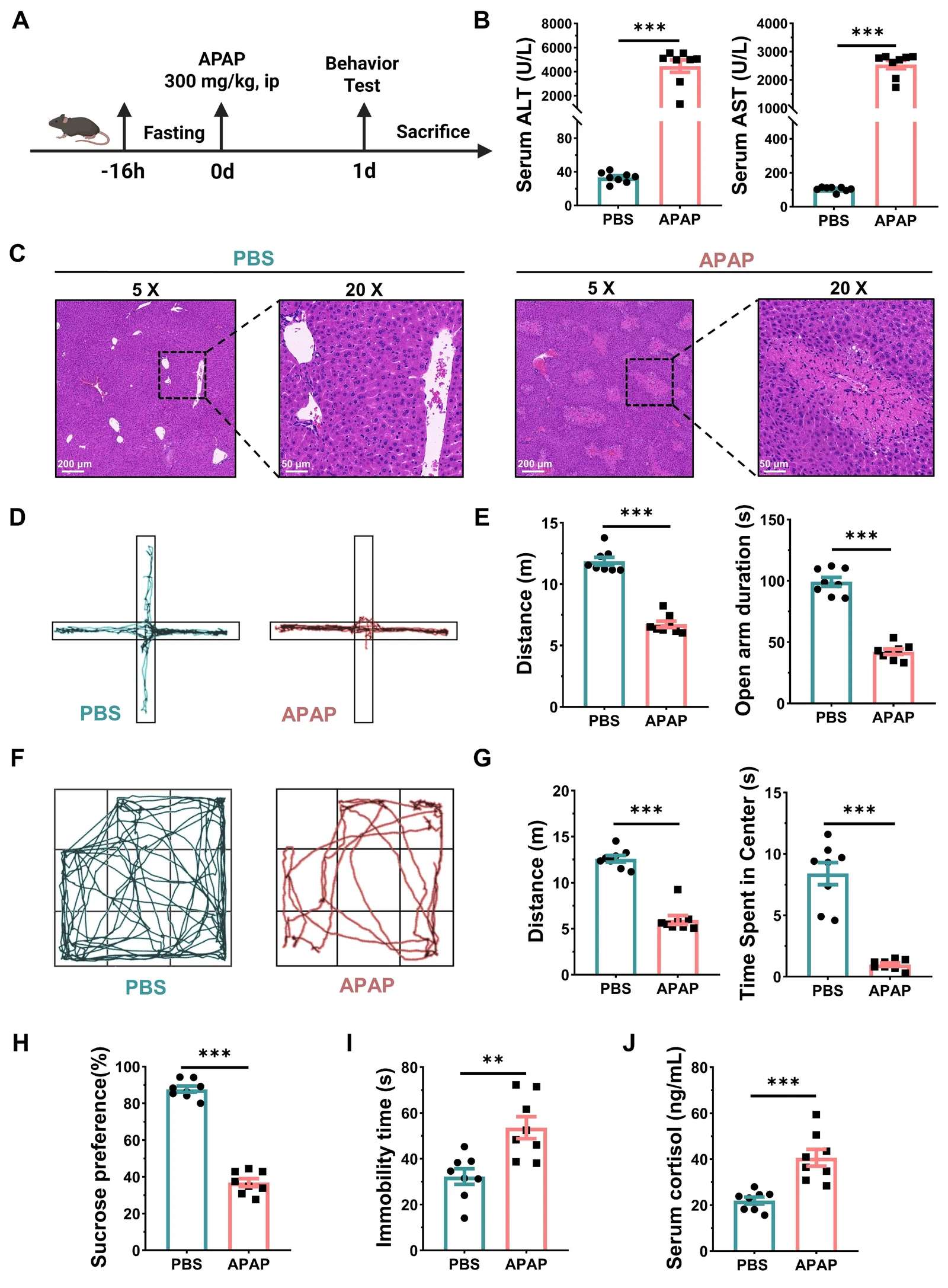
** APAP exposure induces liver injury and leads to anxiety- and depressive-like behaviors.** (A) Scheme of the DILI model and behavioral tests in mice. (B) Statistical results show that APAP exposure increased serum ALT and AST levels (n = 8). (C) Representative H&E staining of liver tissue. Scale bar = 200 µm; scale bar = 50 µm. (D-E) Representative traces and statistical results show that DILI decreased the distance and the open arm duration in the EPM (n = 8). (F-G) Representative traces and statistical results show that DILI decreased the distance and the time spent in the center area in the OFT (n = 8). (H) Statistical results show that DILI decreased sucrose preference in the SPT (n = 8). (I) Statistical results show that DILI increased immobility time in the FST (n = 8). (J) Statistical results show that DILI increased serum cortisol levels (n = 8). Data are expressed as mean ± SEM. **P < 0.01, ***P < 0.001.

**Figure 2:**
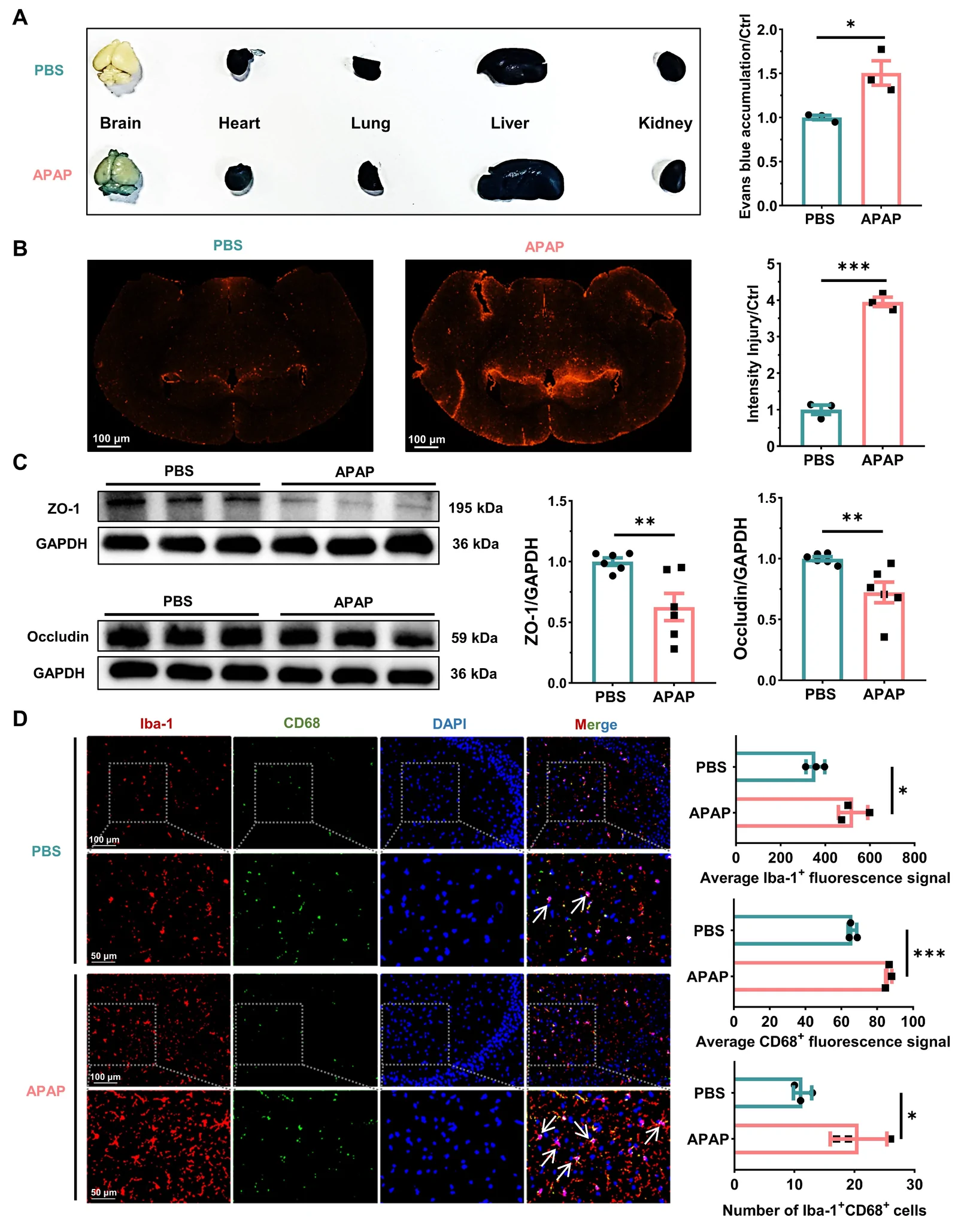
** DILI disrupts the BBB and activates microglia.** (A) Representative images of brains and peripheral organs from DILI after Evans blue administration and statistical results show that DILI increased Evans blue accumulation (n = 3). (B) Representative images and quantification of Evans blue staining of brain tissues show that DILI disrupted the integrity of the BBB (n = 3). Scale bar = 100 µm. (C) Representative immunoreactive bands and statistical results show that DILI decreased the expression of cortical ZO-1 and Occludin (n = 6). (D) Representative images and quantification of Iba-1/CD68 per unit area show that DILI facilitated the colocalization between Iba-1 and CD68 in the hippocampal CA3 (n = 3). Scale bar = 100 µm; scale bar = 50 µm. Data are expressed as mean ± SEM. *P < 0.05, **P < 0.01, ***P < 0.001.

**Figure 3:**
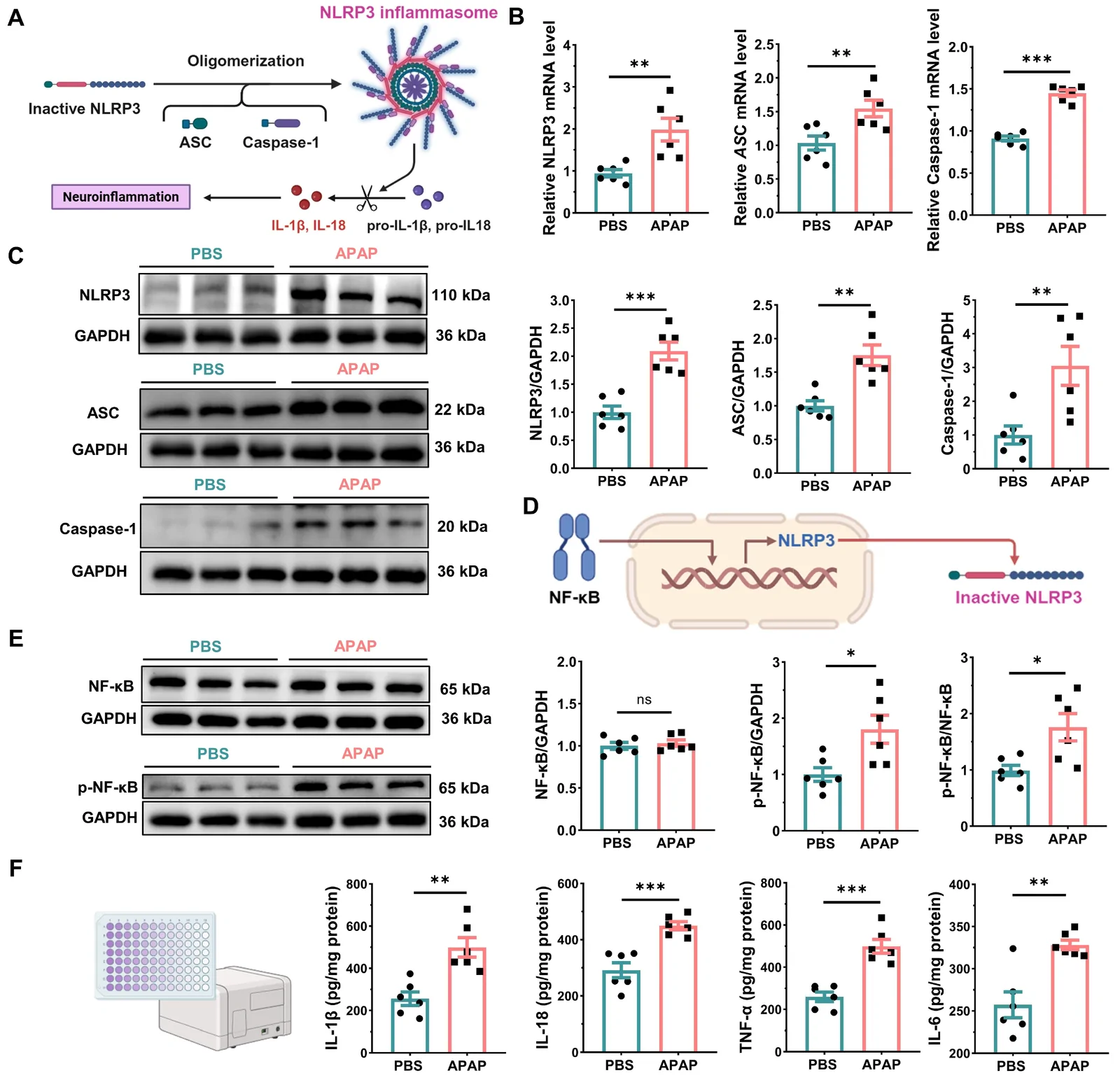
** DILI triggers hippocampal NLRP3 inflammasome activation.** (A) NLRP3 inflammasome activation. (B) Statistical results show that DILI increased the mRNA expression of NLRP3, ASC and caspase-1 in the hippocampus. (C) Representative immunoreactive bands and statistical results show that DILI increased the expression of hippocampal NLRP3, ASC and caspase-1. (D) NF-κB/NLRP3 inflammasome signaling. (E) Representative immunoreactive bands and statistical results show that DILI increased the p-NF-κB/NF-κB ratio in the hippocampus. (F) Statistical results show that DILI increased the levels of IL-1β, IL-18, TNF-α, and IL-6 in the hippocampus. Data are expressed as mean ± SEM. n = 6, *P < 0.05, **P < 0.01, ***P < 0.001.

**Figure 4:**
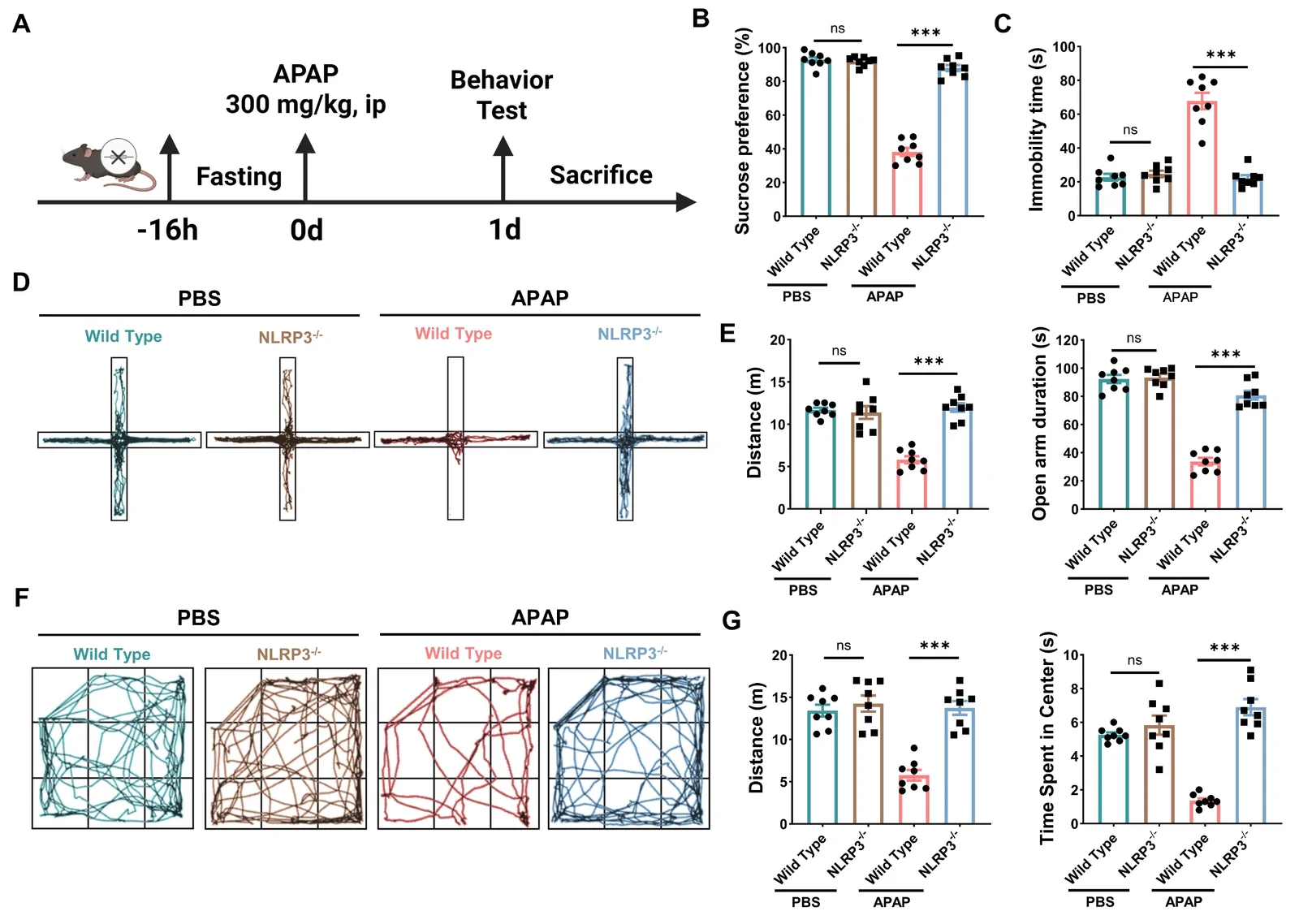
***Nlrp3* knockout alleviates DILI-induced anxiety- and depressive-like behaviors in mice.** (A) Scheme of the DILI model and behavioral tests in *Nlrp3*^-/-^ mice. (B) Statistical results show that *Nlrp3* knockout alleviated DILI-induced decrease in sucrose preference in the SPT. (C) Statistical results show that *Nlrp3* knockout alleviated DILI-induced increase in immobility time in the FST. (D-E) Representative traces and statistical results show that *Nlrp3* knockout alleviated DILI-induced decrease in the distance and the open arm duration in the EPM. (F-G) Representative traces and statistical results show that *Nlrp3* knockout alleviated DILI-induced decrease in the distance and the time spent in the center area in the OFT. Data are expressed as mean ± SEM. n = 8, ***P < 0.001.

**Figure 5:**
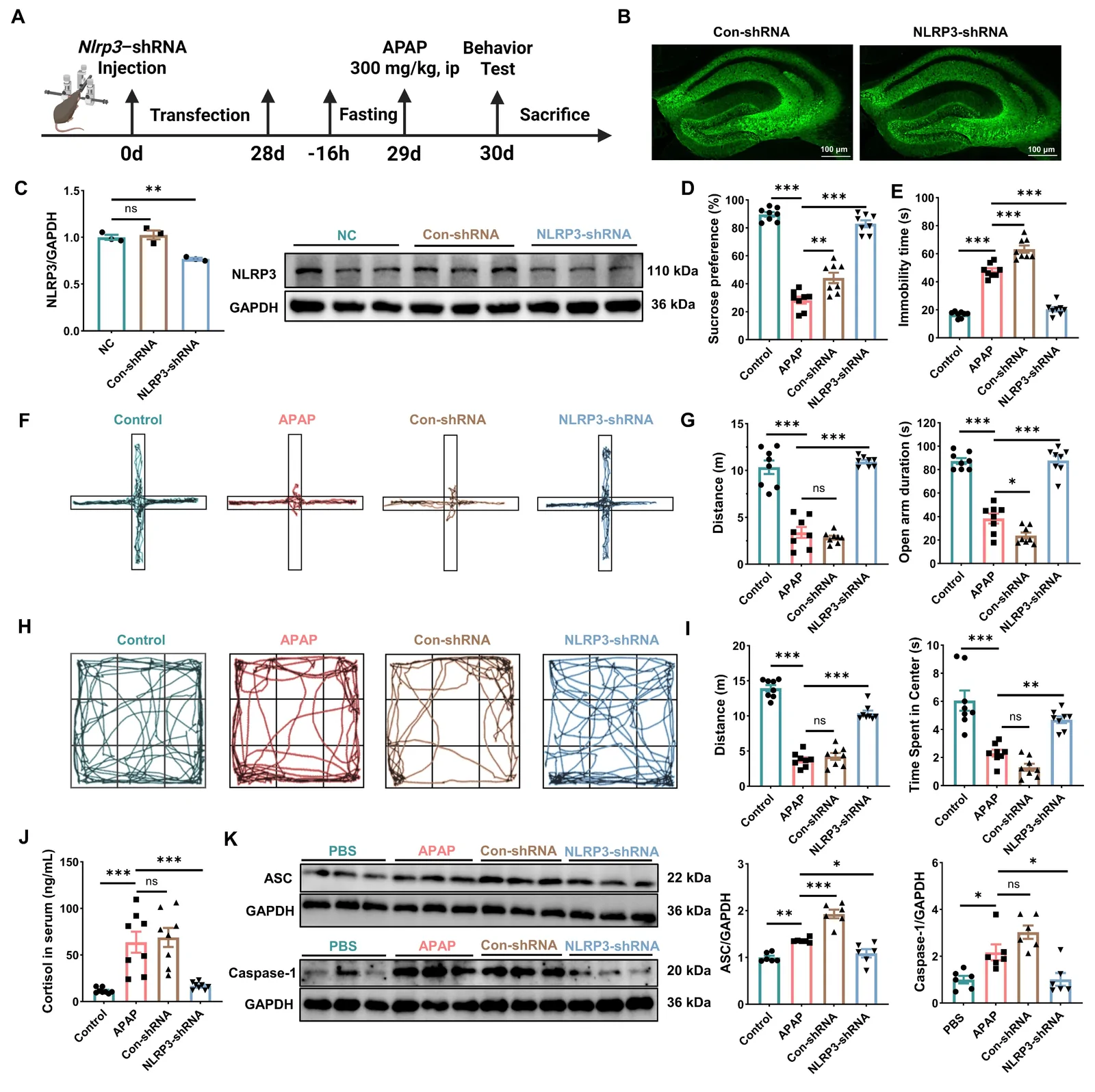
** Hippocampal *Nlrp3* knockdown ameliorates DILI-induced anxiety- and depressive-like behaviors in mice.** (A) Scheme of the DILI model and behavioral tests conducted following AAV-mediated hippocampal *Nlrp3* knockdown. (B-C) Hippocampal immunofluorescence image and representative immunoreactive bands showing the knockdown efficacy of AAV-*Nlrp3*-shRNA (n = 3). Scale bar = 100 µm. (D) Statistical results show that hippocampal *Nlrp3* knockdown alleviated DILI-induced decrease in sucrose preference in the SPT (n = 8). (E) Statistical results show that hippocampal *Nlrp3* knockdown alleviated DILI-induced increase in immobility time in the FST (n = 8). (F-G) Representative traces and statistical results show that hippocampal *Nlrp3* knockdown alleviated DILI-induced decrease in the distance and the open arm duration in the EPM (n = 8). (H-I) Representative traces and statistical results show that hippocampal *Nlrp3* knockdown alleviated DILI-induced decrease in the distance and the time spent in the center area in the OFT (n = 8). (J) Statistical results show that hippocampal *Nlrp3* knockdown alleviated DILI-induced increase in the levels of serum cortisol (n = 8). (K) Representative immunoreactive bands and statistical results show that hippocampal *Nlrp3* knockdown alleviated DILI-induced increase in the expression of hippocampal ASC and caspase-1 (n = 6). Data are expressed as mean ± SEM. *P < 0.05, **P < 0.01, ***P < 0.001.

**Figure 6:**
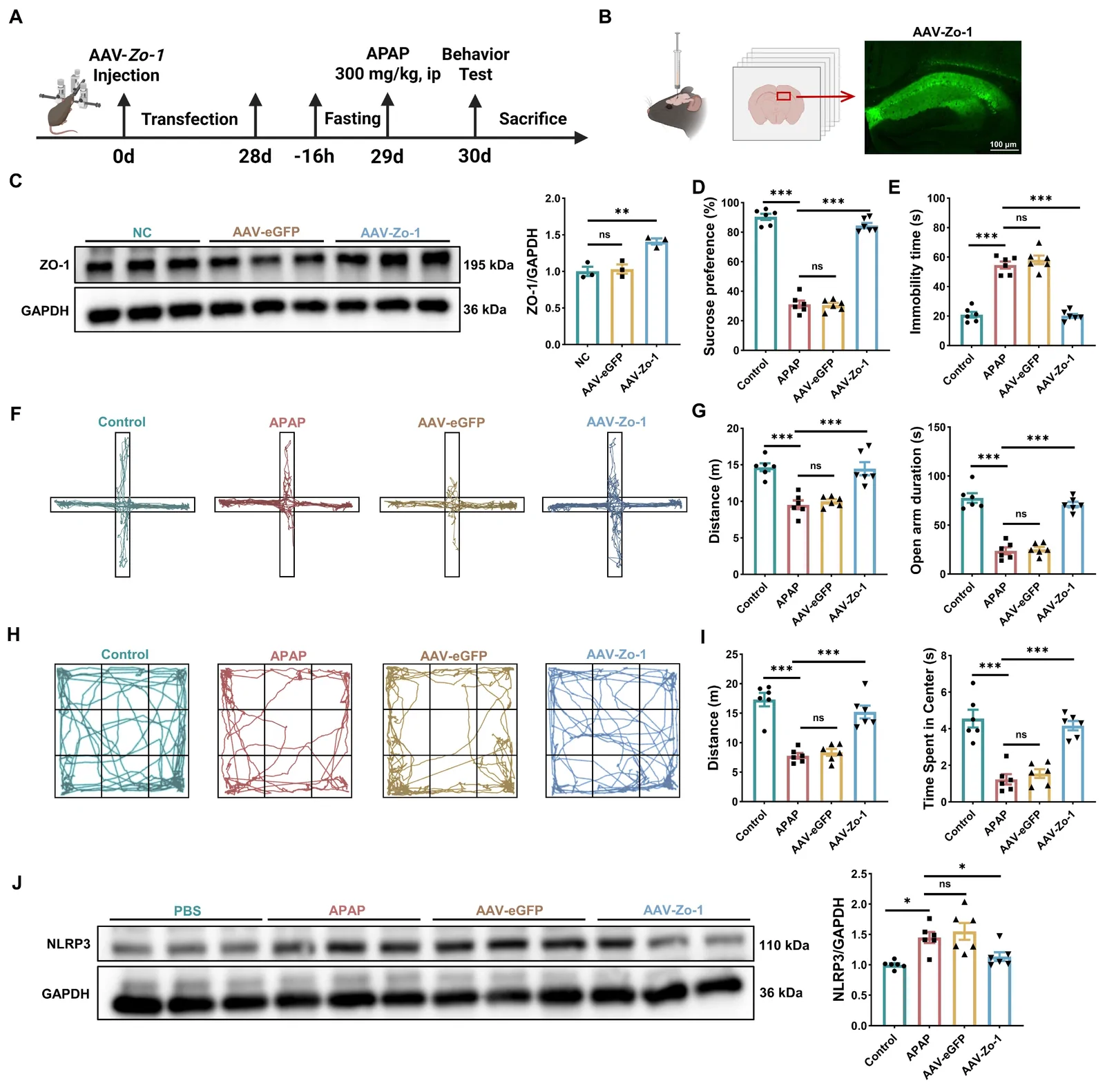
** Targeted BBB protection alleviates DILI-induced anxiety- and depressive-like behaviors in mice.** (A) Scheme of the DILI model and behavioral tests conducted following AAV-mediated *Zo-1* overexpression. (B-C) Immunofluorescence images and representative immunoreactive bands show overexpression efficiency of AAV-*Zo-1* (n = 3). Scale bar = 100 µm. (D) Statistical results show that targeted BBB protection alleviated DILI-induced decrease in sucrose preference in the SPT (n = 6). (E) Statistical results show that targeted BBB protection alleviated DILI-induced increase in immobility time in the FST (n = 6). (F-G) Representative traces and statistical results show that targeted BBB protection alleviated DILI-induced decrease in the distance and the open arm duration in the EPM (n = 6). (H-I) Representative traces and statistical results show that targeted BBB protection alleviated DILI-induced decrease in the distance and the time spent in the center area in the OFT (n = 6). (J) Representative immunoreactive bands and statistical results show that targeted BBB protection alleviated DILI-induced increase in the expression of NLRP3 (n = 6). Data are expressed as mean ± SEM. *P < 0.05, **P < 0.01, ***P < 0.001.

**Figure 7:**
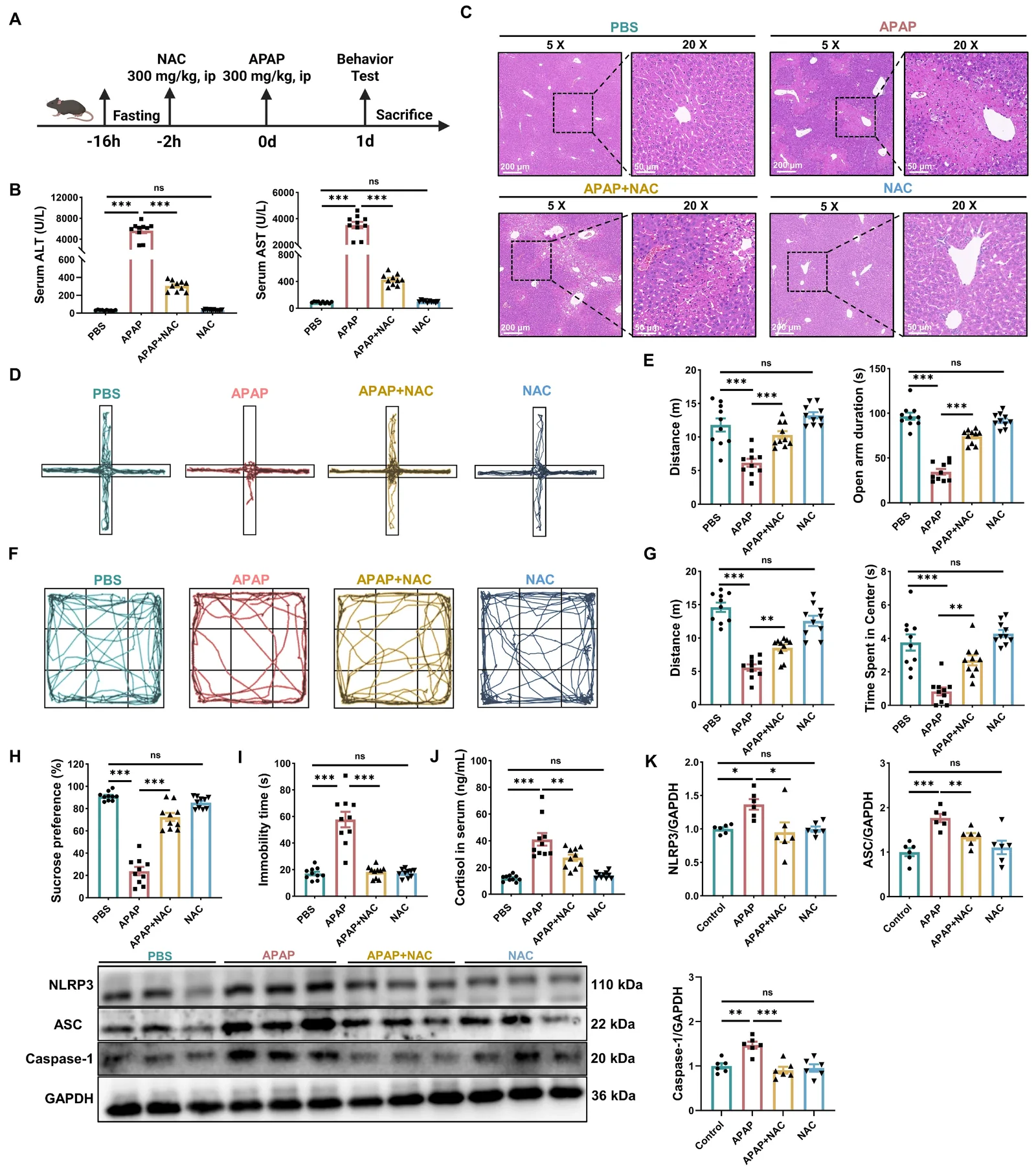
** NAC administration attenuates DILI and alleviates anxiety- and depressive-like behaviors in mice.** (A) Scheme of the DILI model and behavioral tests conducted in NAC-treated mice. (B) Statistical results show that NAC attenuated DILI-induced increase in the levels of serum ALT and AST (n = 10). (C) Representative H&E staining of liver tissues. Scale bar = 200 µm; scale bar = 50 µm. (D-E) Representative traces and statistical results show that NAC alleviated DILI-induced decrease in the distance and the open arm duration in the EPM (n = 10). (F-G) Representative traces and statistical results show that NAC alleviated DILI-induced decrease in the distance and the time spent in the center area in the OFT (n = 10). (H) Statistical results show that NAC alleviated DILI-induced decrease in sucrose preference in the SPT (n = 10). (I) Statistical results show that NAC alleviated DILI-induced increase in immobility time in the FST (n = 10). (J) Statistical results show that NAC alleviated DILI-induced increase in the levels of serum cortisol (n = 10). (K) Representative immunoreactive bands and statistical results show that NAC alleviated DILI-induced increase in the expression of hippocampal NLRP3, ASC and caspase-1 (n = 6). Data are expressed as mean ± SEM. *P < 0.05, **P < 0.01, ***P < 0.001.

## Data Availability

The data sets used and/or analyzed during the current study are available from the corresponding author on reasonable request.

## Abbreviations

AAV: adeno-associated virus
ALT: alanine aminotransferase
APAP: acetaminophen
ASC: apoptosis-associated speck-like protein containing a caspase-recruitment domain [CARD]
AST: aspartate aminotransferase
BBB: blood-brain barrier
Caspase-1: cysteinyl aspartate specific proteinase-1
CNS: central nervous system
CYP2E1: cytochrome P450 2E1
DILI: drug-induced liver injury
EPM: elevated plus maze
FST: forced swimming test
GSH: glutathione
IL-1β: interleukin-1β
IL-18: interleukin-18
IL-6: interleukin-6
LC-MS/MS: liquid chromatography-tandem mass spectrometry
MDD: major depressive disorder
NAC: N-acetylcysteine
NAPQI: N-Acetyl-4-benzoquinone imine
NF-κB: nuclear factor-kappaB
NLR: NOD-like receptor
NLRP3: NLR family, pyrin domain containing 3
OFT: open-field test
SPT: sucrose preference test
TNF-α: tumor necrosis factor-α
