# Supplementary material for: Hippocampal NLRP3 Inflammasome Drives Anxiety- and Depressive-Like Behaviors Following Drug-Induced Liver Injury
Source: Int J Biol Sci. 2026 Sep 3;22(14):7782–99. doi: 10.7150/ijbs.128620 (PMC13618000; doi:10.7150/ijbs.128620)
Supplement: Supplementary file 1 [file ijbsv22p7782s1.pdf]

Supplementary figures

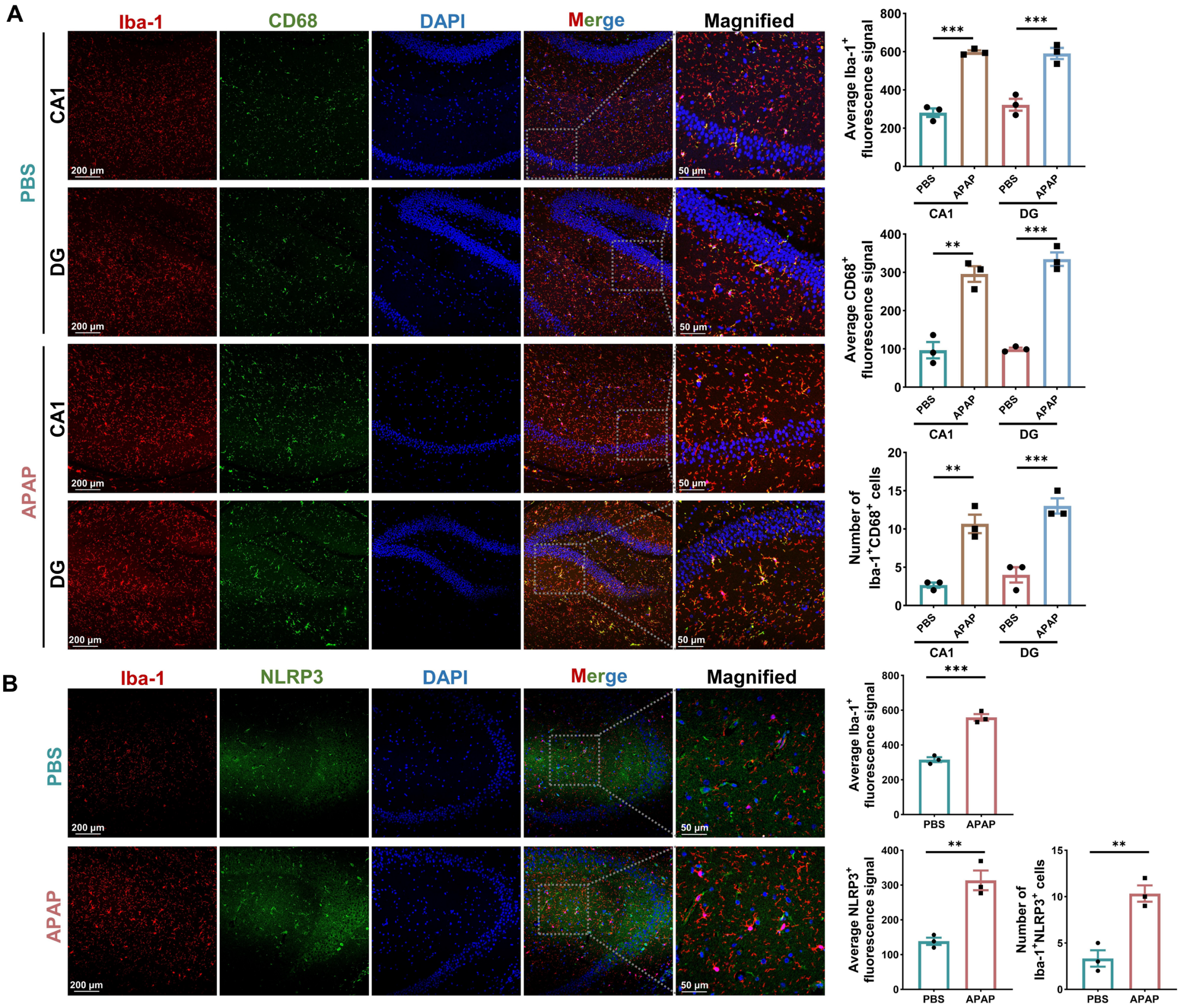

Figure S1. DILI induces microglial activation and increases Iba-1/NLRP3 colocalization in the hippocampus. (A) Representative images and quantification of Iba-1/CD68 per unit area show that DILI facilitated the colocalization between Iba-1 and CD68 in the hippocampal CA1 and DG. (B) Representative images and quantification of Iba-1/NLRP3 per unit area show that DILI facilitated the colocalization between Iba-1 and NLRP3 in the hippocampal CA3. Scale bar = 200 μm; scale bar = 50 μm. Data are expressed as mean ± SEM. n = 3, $^{**}P < 0.01$, $^{***}P < 0.001$.

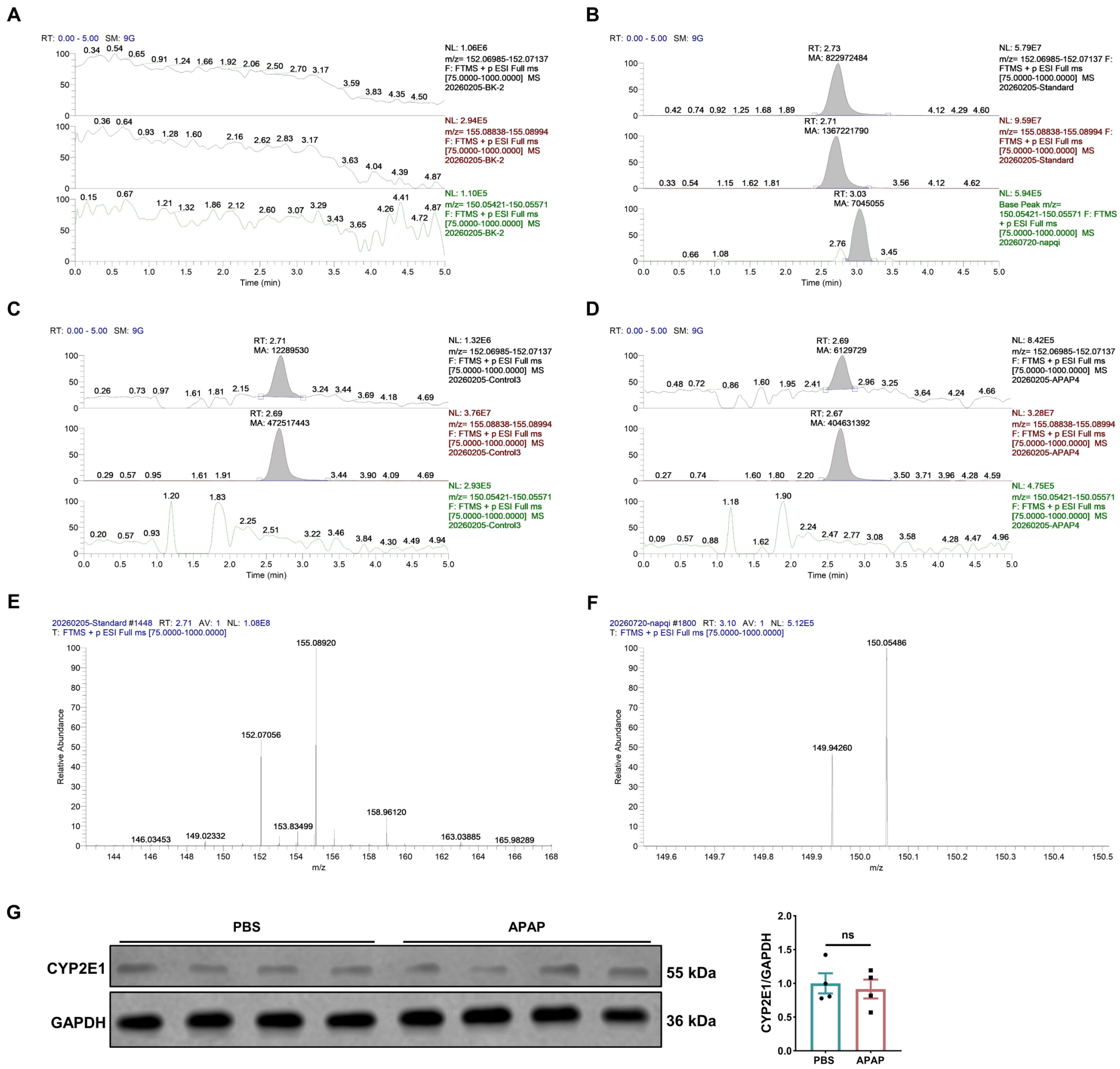

Figure S2. Qualitative analysis of APAP and NAPQI in brain tissue. (A) EIC chromatogram of blank solvent group (The upper, middle, and lower panels show the EICs for APAP, APAP-d_3, and NAPQI, respectively). (B) EIC chromatogram of reference standard group (The upper and lower panels show the EICs for the APAP and NAPQI reference standards, respectively, while the middle panel shows the internal standard APAP-d_3). (C) EIC chromatogram of PBS group (The upper and lower panels show the levels of APAP and NAPQI in the PBS group, respectively, with the internal standard APAP-d_3 presented in the middle panel). (D) EIC chromatogram of APAP group (The upper and lower panels show the levels of APAP and NAPQI in the APAP group, respectively, with the internal standard APAP-d_3 presented in the middle panel). (E) The high-resolution mass spectrum of the reference standard shows the APAP ion at m/z 152.07056 and the APAP-d_3 ion at m/z 155.08920. (F) The high-resolution mass

spectrum of the NAPQI reference standard shows the NAPQI ion at m/z 150.05486. (G) Representative immunoreactive bands and statistical results demonstrated that DILI did not significantly alter CYP2E1 expression in the hippocampus. Data are expressed as mean ± SEM. n = 4, ns, not significant.

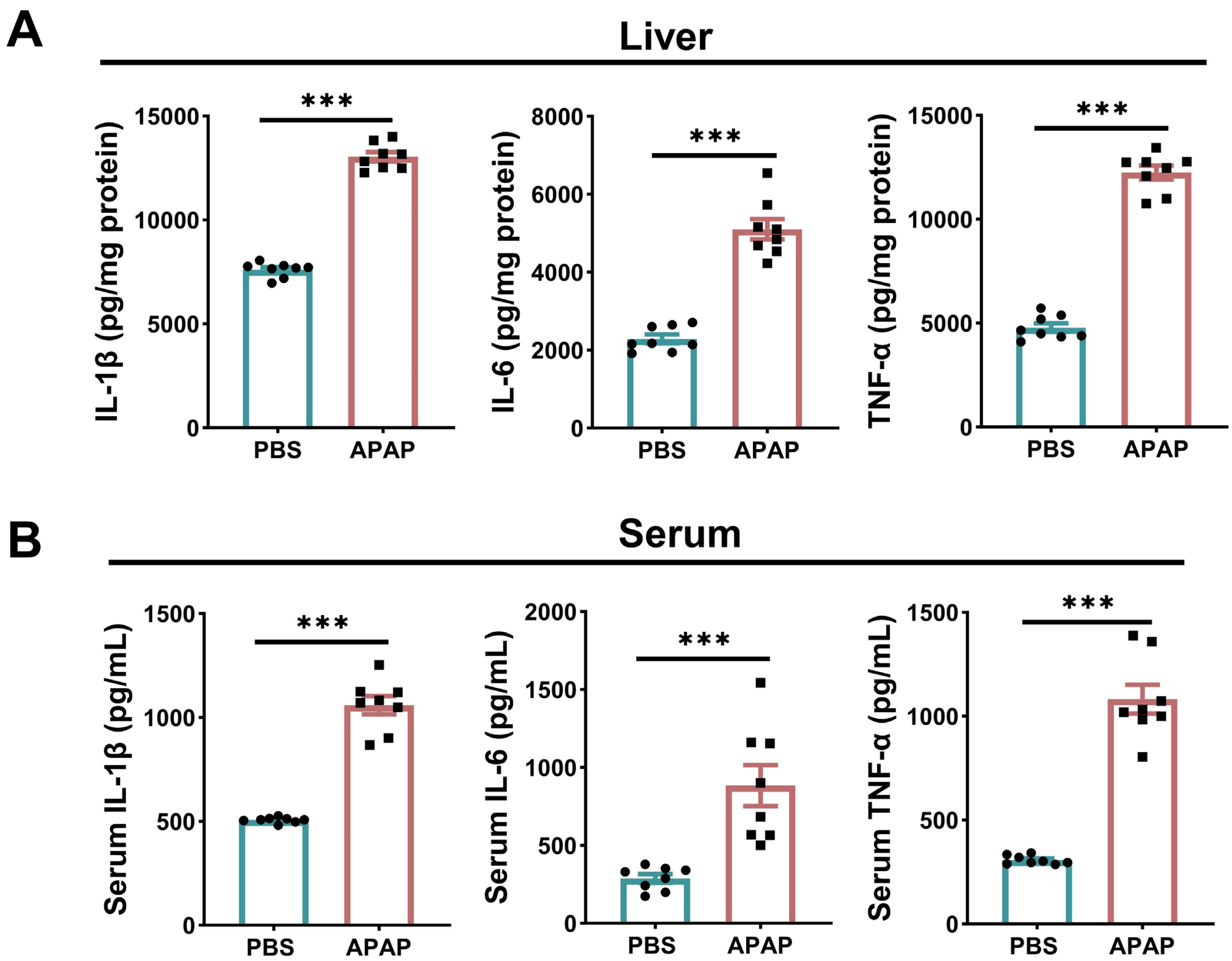

Figure S3. DILI can lead to an increase in proinflammatory factors in both the liver and circulation. (A) Statistical results show that DILI increased the levels of IL-1β, IL-6, and TNF-α in the liver. (B) Statistical results show that DILI increased the levels of IL-1β, IL-6, and TNF-α in the circulation. Data are expressed as mean ± SEM. n = 8, ***$P < 0.001$.

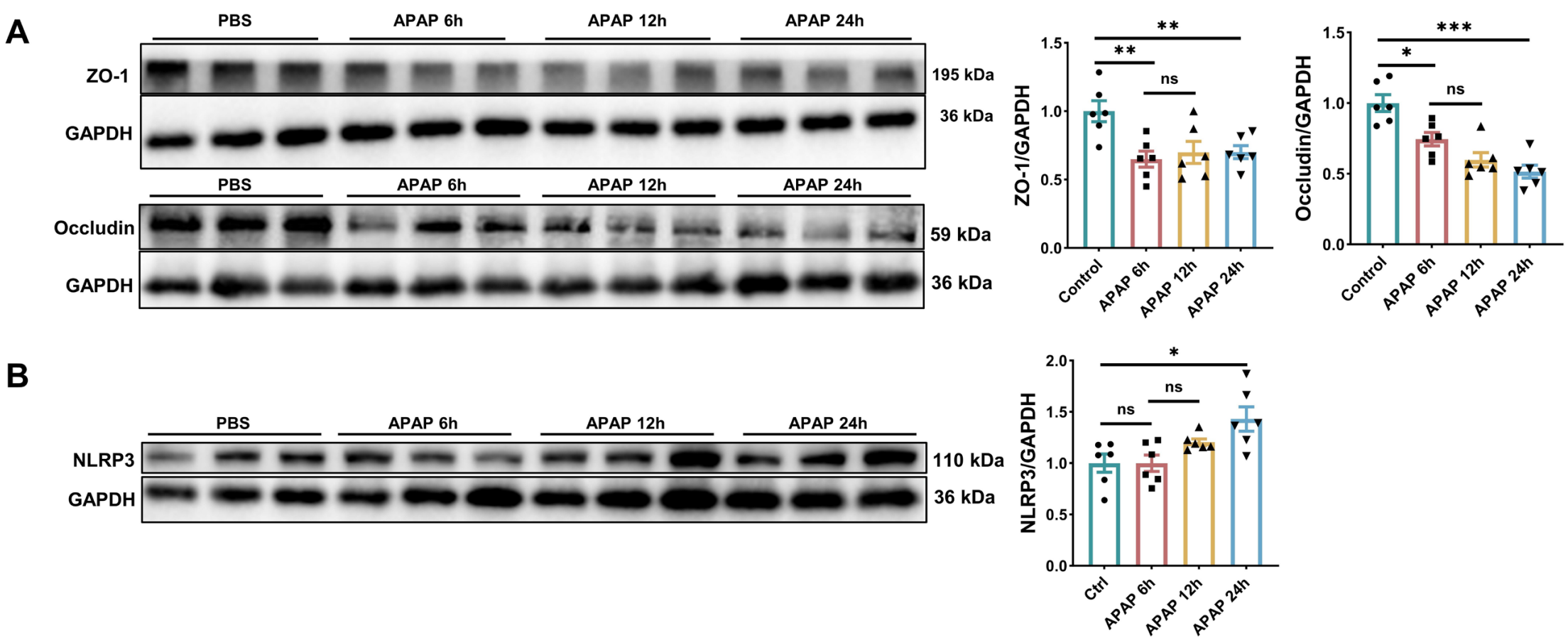

Figure S4. Time-course analyses suggest BBB destruction precedes NLRP3 activation in DILI. (A) Representative immunoreactive bands and statistical results revealed that the expression of ZO-1 and Occludin in the cortex was significantly decreased at 6, 12, and 24 h following APAP injection. (B) Representative immunoreactive bands and statistical results show that expression of NLRP3 in the hippocampus was significantly increased at 6, 12, and 24 h following APAP injection. Data are expressed as mean ± SEM. n = 6, *P < 0.05, **P < 0.01, ***P < 0.001.

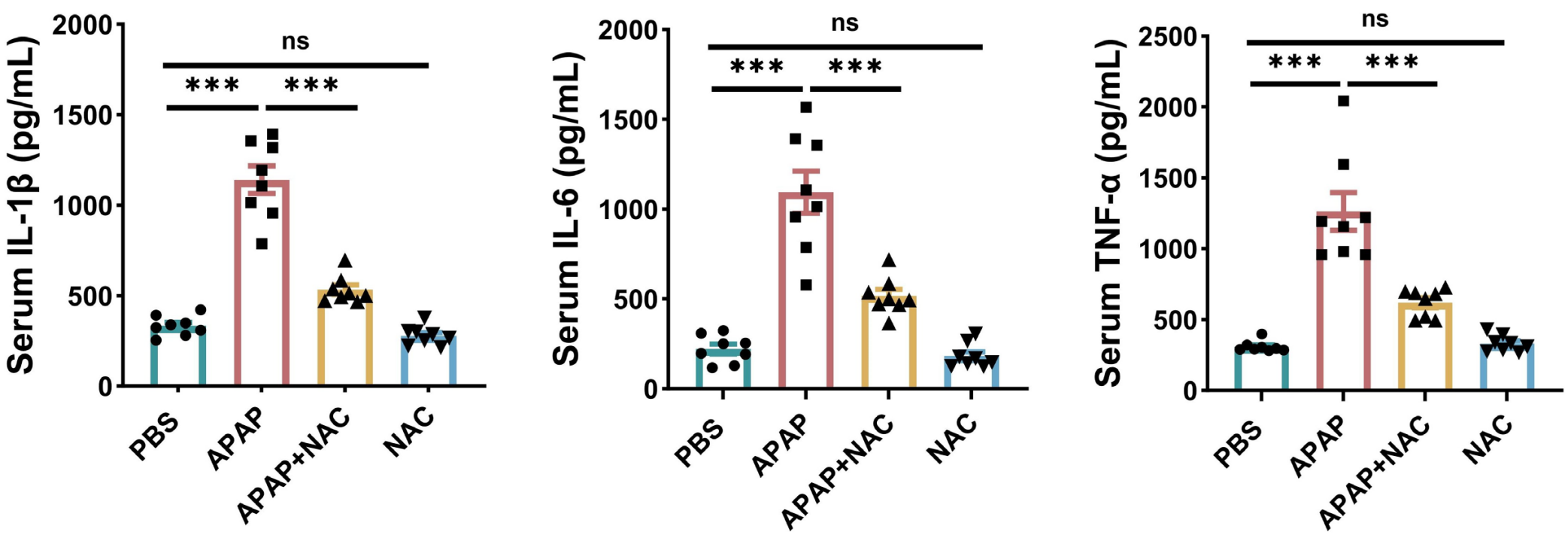

Figure S5. NAC attenuates DILI-induced elevations of circulating proinflammatory cytokines. Statistical analysis demonstrate that NAC significantly attenuated the DILI-induced increases in circulating levels of IL-1β, IL-6, and TNF-α. Data are expressed as mean ± SEM. n = 8, ***P < 0.001.
